# Obesity, Serum Resistin and Leptin Levels Linked to Coronary Artery Disease

**DOI:** 10.5935/abc.20160134

**Published:** 2016-10

**Authors:** Farzaneh Montazerifar, Ahmad Bolouri, Raheleh Sharifian Paghalea, Mahbubeh Khodadadpour Mahani, Mansour Karajibani

**Affiliations:** 1 Pregnancy Health Research Center - Departament of Nutrition - School of Medicine - Zahedan University of Medical Sciences; Zahedan, Irã; 2 Departament of Cardiology School of Medicine - Zahedan University of Medical Sciences; Zahedan, Irã; 3 School of Medicine - Zahedan University of Medical Sciences; Zahedan, Irã; 4 Health Promotion Research Center - Departament of Nutrition - School of Medicine - Zahedan University of Medical Sciences, Zahedan, Irã

**Keywords:** Coronary Artery Disease, Obesity, Resistin, Leptin, Atherosclerosis

## Abstract

**Background:**

Clinical studies have demonstrated that adipocytokines play an important role in developing atherosclerotic cardiovascular diseases.

**Objective:**

The aim of study was to evaluate the relationship between serum resistin and leptin levels with obesity and coronary artery disease (CAD).

**Methods:**

In a cross-sectional study, we assessed the levels of serum resistin and leptin, C-reactive protein (CRP), lipid profile and cardiac enzyme tests (AST, CPK, LDH, CK-MB) in 40 CAD patients compared to 40 healthy controls. Anthropometric measurements including weight and height for calculating of body mass index (BMI), and waist circumference (WC) were performed for evaluation of obesity.

**Results:**

CAD patients had increased levels of leptin and CRP, (p < 0.001), cholesterol (p < 0.05), triglyceride (p < 0.01), and WC (p < 0.05) compared to healthy controls. There was no statistical difference between CAD and control subjects for resistin (p = 0.058). In a multiple regression analysis, only an association between serum leptin with BMI (β = 0.480, p < 0.05) and WC (β = 1.386, p < 0.05) was found.

**Conclusions:**

The findings suggest that leptin is a better marker of fat mass value than resistin and may be considered an independent risk factor for cardiac disorders that is largely dependent on obesity. However, further prospective studies are needed to confirm these results.

## Introduction

Obesity and coronary artery disease (CAD) are the most important health problems worldwide, especially in the adult Iranian population.^1-3^ CAD is one of the major atherosclerotic manifestations, and is associated with clinical demonstrations of acute coronary syndrome including angina and myocardial infarction.^1^ Obesity, the most important nutritional disorder in industrialized countries, is a prominent risk factor for CAD.^4-6^ Evidence shows that some forms of obesity, particularly elevated abdominal adiposity, might be responsible for metabolic disorders and vascular diseases.^4,7,8^ The distribution of regional fat, especially the amount of visceral fat around the heart, may affect coronary arteries and the myocardium,^9^ which may be considered a predictive factor for cardiovascular risk.^10^

Clinical studies have demonstrated that apart from classic risk factors such as hypertension, dyslipidaemia, and insulin resistance,^11^ adipocytokines also play an important role in developing atherosclerotic cardiovascular diseases.^1,7,11^ Adipose tissue, abundantly represented in obese rodents and humans, secretes some hormones, peptides, and other molecules that may potentially act as pro-atherogenic markers.^4,8,9,12^ Resistin and leptin, vasoactive substances produced by adipocytes, are potential mediators,^7,12-14^ which contribute to the inflammatory processes related to obesity in both vascular and non-vascular tissues,^4,15,16^ and activate endothelial cells.^15,17,18^ Because of these properties, resistin and leptin have been hypothesized to be a causal factor in the development of cardiovascular diseases, especially atherosclerotic coronary artery disease and congestive heart failure (CHD).^14,16^ Studies on adipokines and obesity have shown that elevated levels of resistin^19,20^ and leptin^14,21-24^ are linked to increased body mass index (BMI), and their receptors are increased in abdominal fat depots.^4^ Due to the high prevalence of CAD, the evaluation of serum levels of adipokines may be used as a prognostic marker in screening, diagnosing and predicting atherosclerosis. Thus, we designed this study to investigate the relationship between changes in resistin and leptin levels with obesity and CAD.

## Methods

### Study patients

In a case-control study, forty patients aged 30-80 years old (mean age of 55.6 ±13.4 yr; BMI of 25 ± 4.8 kg/m^2^) admitted to the cardiology section of Emam Ali hospital of Zahedan , Iran, who had 50% or more coronary stenosis in at least one major coronary artery were enrolled in the CAD group. Exclusion criteria included medical history (e.g., diabetes mellitus, thyroid , liver or renal failure , cardiomyopathy, left ventricular systolic dysfunction or severe heart failure, acute or chronic inflammatory disorders, or the recent use of lipid-lowering drugs and corticosteroids or smoking). After matching for age and sex, 40 healthy volunteers aged 30-79 years old (mean age of 53 ±12 yr; BMI of 25.7± 4.9 kg/m^2^) without cardiovascular and any organ system disease and on no medications were selected as the control group. The study was performed between June and December of 2014.

### Methodology

A demographic questionnaire, including age, sex, BMI, waist circumference (WC), medical history including smoking habit, presence of hypertension, hyperlipidemia and current medications was filled out by all subjects. Simple anthropometric measurements including weight and height to calculate BMI, and WC were performed for evaluation of obesity.^25^ The measurements of weight and height were performed with light clothing and without shoes, and approximated to the nearest 0.5 kg and 0.5 cm, respectively. The WC was measured with a non-stretchable standard tape, at the narrowest point between the costal margin and iliac crest. BMI ≥ 25 kg/m^2^ (general obesity), and WC > 102 cm in men and > 88 cm in women (abdominal obesity) were considered risk factors for cardiovascular disease.^4,25^

Blood samples were taken after a 12-hour overnight fast. Biochemical parameters including serum cholesterol, triglyceride, high-density lipoprotein cholesterol (HDL), low-density lipoprotein cholesterol (LDL), creatine phosphokinas (CPK), lactate dehydrogenase (LDH), creatine kinase (CK-MB), and aspartate transaminase (AST) levels were measured by commercial kits (Pars azmun ,Tehran, Iran) using an auto-analyzer (Hitachi , Japan). Serum high-sensitivity C-reactive protein (hs-CRP) levels were assessed by latex- enhanced nephelometry (Behring BN II nephelometer, Germany).

Levels of serum resistin and leptin were measured by enzyme-linked immune-sorbent assay (ELISA) with commercial kits: [HUMAN resistin ELISA kit (Cat.No:EK0581; Boster biological technology; 40459 Encyclopedia Circle, Fermont, CA 94538, USA], and [HUMAN leptin ELISA kit (Cat. No: RD 191001100, USA).The serum samples were immediately frozen at -70ºC until analysis.

The study was approved by the ethics committee of (omitted to the review process) and informed consent was obtained from all subjects. (Approval date: 21 April 2014; Code No: 6696).

### Statistical analysis

The analysis was performed by SPSS statistical software package program (version 18 for windows, Chicago, USA). Data were tested for normal distribution using the Kolmogorov- Smirnov test. Data were expressed as mean ± SD or mean ± SEM in accordance with their distribution. Variables with normal distribution were compared by unpaired Student's t*-*test and one-way ANOVA. Mann-Whitney U test was performed for non-normal distribution variables. Resistin and leptin values were compared using multivariable regression analysis, adjusted for age and sex. P value < 0.05 was considered significant.

## Results

Demographic and chemical characteristics of subjects have been summarized in Table 1. Age, BMI, LDL and HDL levels were not significantly different between patients and controls. The mean WC (p < 0.05), serum cholesterol (p < 0.05), triglyceride (p < 0.01) and hs-CRP (p < 0.001) levels were markedly increased when compared to healthy controls.

Compared to the controls, serum levels of leptin were significantly higher in CAD patients (p < 0.001). Serum resistin levels differed between two groups, but this difference was not significant (p = 0.058).

**Table 1:** Demographic and chemical characteristics of subjects

	CAD Patients (n = 40)	Controls (n = 40)	p
Age (yr)	55.6 ± 13.4	53 ± 12	NS
Sex (M/F)	20/20	23/17	
BMI (Kg/m^2^)	25 ± 4.8	25.7 ± 4.9	NS
**WC(Cm)**			
Male	96.8 ± 13.5	86.1 ± 12.5	0.05
Female	91.7 ± 12.7	88.9 ±15.4	0.05
Cholesterol (mg/dL)	193.5 ± 49.6	169 ± 42.6	0.05
LDL-C (mg/dL)	89 ± 29.7	82.3 ± 23.7	NS
HDL-C (mg/dL)	42 ± 13.4	43.2 ± 12	NS
Triglyceride (mg/dL)	181 ± 94	119 ± 79.4	0.01
*AST (U/L)	97.6 ± 21.3	26 ± 7.4	0.001
*LDH (U/L)	711 ± 101.2	191 ± 40	0.0001
*CPK (U/L)	637.4 ± 195.6	97.2 ± 49	0.0001
*CK-MB (U/L)	81.5 ± 28	17 ± 4.1	0.001
*CRP (mg/L)	13.4 ± 3.5	1.3± 0.09	0.001
Resistin (ng/mL)	2.6 ± 1	2.1 ± 0.8	0.058
*Leptin (ng/mL)	41.7 ± 6.5	24.1 ± 2.9	0.001
	(1.2 - 196)	(1.5 - 86)	

Data are presented as mean ± SD.

* Leptin, Cardiac enzyme tests (ALT, CPK, LDH, CK-MB) and CRP levels in patients are presented as mean ± SEM, because the data were not normally distributed. BMI: body mass index; WC: waist circumference; LDL: low density lipoprotein; HDL: high density lipoprotein; ALT: aspartate transaminase; CPK: creatine phosphokinas; LDH: lactate dehydrogenize; CK-MB: creatine kinase -MB; CRP: C-reactive protein; NS: not significant.

**Figure 1:**
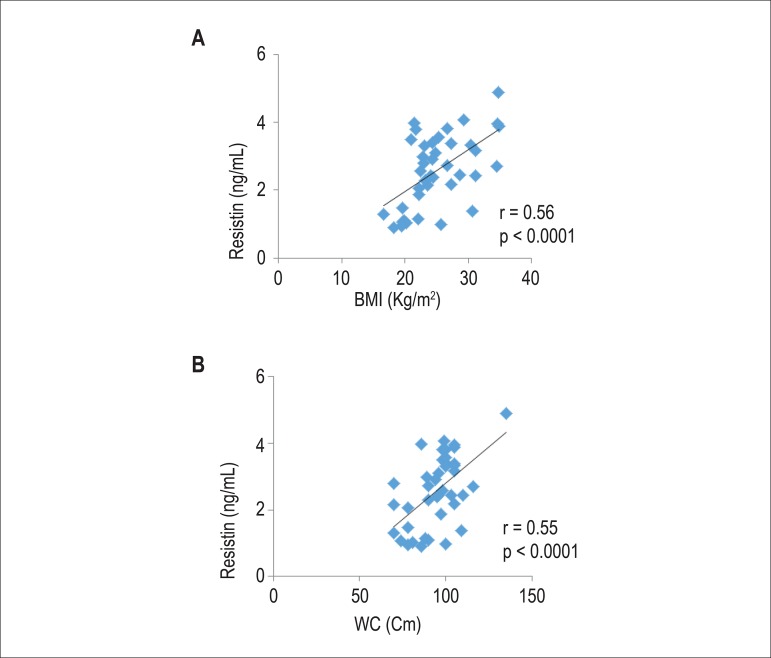
Correlation between resistin with body mass index (BMI) (A) and waist circumference (WC) (B).

**Figure 2:**
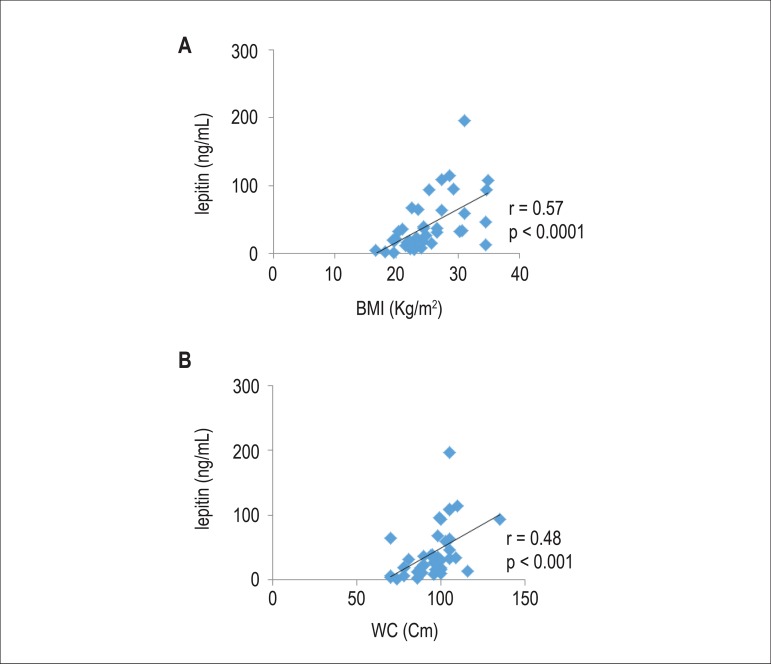
Correlation between leptin with body mass index (BMI) (A) and waist circumference (WC) (B)

As shown in Figures1 and 2, a positive correlations between resistin and BMI (r = 0.56, p < 0.0001) and WC (r = 0.55, p < 0.0001), and between leptin with BMI (r = 0.57, p < 0.0001) and WC (r = 0.48, p < 0.001) were found.

In multiple regression analysis, leptin was associated with BMI ((β = 0.480, p < 0.05) and WC (β = 1.386, p < 0.05) in CAD patients, but this association was not significant for resistin.

## Discussion

The present study evaluated the relationship between resistin and leptin levels with obesity and some risk factors of CAD. We demonstrated that the serum resistin concentration differed between CAD patients and the control group, but this difference was not significant, confirming results of previous studies.^18,25^ Several studies have reported serum resistin levels to be significantly elevated in CAD patients.^4,19,26,27^ In contrast, other studies^16,28^ found no such correlation. In clinical and experimental studies, resistin has been suggested to be an independent inflammatory marker in cardiovascular diseases, especially in CAD and heart failure.^16,28-30^

High serum leptin levels observed in patients with CAD in our study were consistent with earlier studies,^22,31,32^ suggesting the role of this hormone as a mediator in human atherosclerotic. By contrast, some data indicate that leptin may protect against atherosclerosis in specific animal models,^33^ and a study^15^ found no significant difference between CHD patients and controls.

Regarding BMI and WC findings, although BMI is recognized as a gold standard indicator for evaluation of obesity,^4,34^ it is not always a reliable measurement of body composition, because it cannot show the regional distribution of body fat. In fact, people with similar body mass indexes may have different amounts of fat in the bodies. Thus, WC was measured to determine visceral fat accumulation, and as an indicator of health risks associated with central obesity.^4^ Recent studies suggest that the central (abdominal, visceral) distribution of fat, particularly abdominal fat accumulation, which is a source of pro-inflammatory adipokines, has a more important role in the determination of risk.^4,7,8^ In the present study, after adjustment for sex, age, BMI and WC, obese patients with elevated BMI showed higher serum leptin levels compared to non-obese patients, but this difference was not significant when compared to the control group for resistin levels (data not shown). As well, resistin and leptin levels were significantly higher in abdominal obese patients than in patients without abdominal obesity or in the control group. Moreover, in multivariate regression analysis, we found an association between serum leptin with BMI and WC after adjusting of age and sex, but this association was not significant for resistin. Our findings were partly consistent with earlier reports,^16,19,29^ but not with some studies.^21,26^ It is worth noting that human resistin is more predominantly expressed in macrophages than adipocytes.^12^ Our findings suggest that leptin is a better marker of fat mass values.

Obesity is also linked to with several established risk factors of cardiovascular disease.^7,8^ Dyslipidemia is one of the most prevalent of CVD risk factors in obesity, especially in abdominal obesity.^35^ Several investigations have reported that dyslipidemia is one of the strongest factors independently associated with CAD in the Iranian population.^1-3,36^ When lipids accumulate within the cells of the arterial wall, it leads to systemic inflammation and atherosclerosis.^4,37^ In this study, we found no significant correlation between resistin and leptin with lipid profile, supporting results of other studies.^15,17^ In some studies, a significant positive association between serum resistin^16,38^ and leptin^15^ with triglyceride and cholesterol levels has been revealed. The reasons for inconsistencies between our findings and other studies may be explained by the study design and sample size.

It has been recently suggested that obesity is related to subclinical inflammation, as reflected by increased CRP levels.^26,39,40^ Evidence shows that resistin and leptin levels with inflammatory activity might play an important role in the development of inflammatory mechanisms and promote the progression of atherosclerotic disease.^7,14,16,20,39^ CRP is one of the best standardized markers for prediction of systemic inflammation degree.^16,40^ Resistin^16,20,26^ and leptin^15,39^ stimulate the production of CRP in coronary endothelial cells, and CRP induces vascular thrombosis that might be involved in the pathophysiology of acute coronary syndromes. In our study, no correlation of CRP was present in CAD patients with resistin and leptin, BMI and WC, suggesting that leptin and resistin are linked to CAD risk regardless of CRP.

Our study had several limitations, including a relatively small sample size. Because of the high cost of resisitin and leptin kits, only 80 subjects (patients and healthy volunteers) were enrolled in our study. Moreover, it was a cross-sectional design and did not prove causation. Therefore, the generalizability of our findings across social and ethnic groups is unknown.

## Conclusion

The study indicated that circulating leptin levels, but not resistin levels, were higher in CAD patients in comparison to controls. As well, in the multivariate regression analysis, after adjusting of age and sex, only an association between serum leptin with BMI and WC was found. It suggests that leptin is a better marker of fat mass value than resistin and may be considered an independent risk factor for cardiac disorders that is largely dependent on obesity. However, further prospective studies are needed to confirm these results.
